# No impact of early intervention on late outcome after minimal, mild and moderate head injury

**DOI:** 10.1186/1757-7241-18-10

**Published:** 2010-02-24

**Authors:** Ben Heskestad, Knut Waterloo, Roald Baardsen, Eirik Helseth, Bertil Romner, Tor Ingebrigtsen

**Affiliations:** 1 Department of Neurosurgery, Stavanger University Hospital, Stavanger, Norway; 2 Department of Neurosurgery, Oslo University Hospital-Ulleval, Oslo, Norway; 3 Department of Neurosurgery, University Hospital of North Norway, Tromsø, Norway; 4 Department of Psychology, University Hospital of North Norway, Tromsø, Norway; 5 Institute for Clinical Medicine, Faculty of Health Sciences, University of Tromsø, Tromsø, Norway

## Abstract

**Objectives:**

To evaluate the effect of an educational intervention on outcome after minimal, mild and moderate head injury.

**Methods:**

Three hundred and twenty six patients underwent stratified randomization to an intervention group (n = 163) or a control group (n = 163). Every second patient was allocated to the intervention group. Participants in this group were offered a cognitive oriented consultation two weeks after the injury, while subjects allocated to the control group were not. Both groups were invited to follow up 3 and 12 months after injury.

**Results:**

A total of 50 (15%) patients completed the study (intervention group n = 22 (13%), control group n = 28 (17%), not significant). There were no statistically significant differences between the intervention group and the control group.

**Conclusions:**

There was no effect on outcomes from an early educational intervention two weeks after head injury.

## Introduction

Minimal, mild and moderate head injuries are common and many patients suffer from post-concussion symptoms after the head injury. Headaches, vertigo, irritability, fatigue, depression and daytime sleepiness are frequent symptoms, but others can be listed. Although post-concussional symptoms usually resolve within days or weeks, mild head injury may have a persistent long-term impact comprising physical, cognitive and emotional sequela for several months or years post injury [1-11].

A number of treatments, including medication for headache, bed rest, and different educational and reassuring strategies, have been suggested as possible preventive measures in observational studies [12]. There is a high prevalence of complaints in the general population, and observational studies in head-injured populations have therefore been criticized. During the last 10 years, five randomized studies of different management strategies have been published [13-17]. The results are conflicting. Studies by Wade et al.[13] suggest that early intervention by a specialist service reduce post-concussion symptoms, while the report by Paniak et al.[14] indicated that a single brief educational intervention delivered soon after head injury was as effective as more intensive regimen of assessment and education. A recent Swedish randomized study showed no significant effect of early intervention in patients with mild traumatic head injury [17].

In the present study, patients were randomized to a single educational intervention two weeks after minimal, mild or moderate head injury, or to no intervention, and thereafter invited to a follow-up 3 and 12 months after the injury. The aim was to study the effect of the educational intervention on outcome.

## Materials and methods

### Participants

The University Hospital of Stavanger is a local hospital for about 300,000 inhabitants. In 2003, a prospective observational study of head injury epidemiology registered a total of 581 referrals for head injury. Head injury was defined as physical damage to the brain or skull caused by external force, and the injuries were classified as minimal, mild, moderate or severe according to the Head Injury Severity Scale [18]. Patients with isolated injuries to the scalp, face or cervical spine were not included. The present report includes all patients with minimal, mild and moderate head injury 15 years and older (n = 326).

Patients were examined and managed according to the Scandinavian guidelines for management of minimal, mild and moderate head injuries [19]. All patients received standardized written information and advice on possible problems and the expected course of improvement after mild head injury at discharge. Age, sex, results from neurological examination including Glasgow Coma Scale (GCS) score, hospital admission or outpatient management and the use of and results from computer tomography (CT) examination were registered.

The 326 included patients underwent stratified randomization to an intervention group (n = 163) or a control group (n = 163). The stratification was done by a study assistant who received consecutive administrative information on included patients. Every second patient was allocated to the intervention group. Participants in this group were offered an educational consultation two weeks after the injury, while subjects allocated to the control group were not. Both groups were invited to follow up 3 and 12 months after injury. A total of 50 (15%) patients completed the study (intervention group n = 22 (13%), control group n = 28 (17%), not significant). The drop out rate was 85%.

### The intervention

The patients in the intervention group were seen by a neurosurgeon or a neurosurgical trainee at the out patient clinic 12-17 days after injury. The standardized written information given to all patients at discharge was orally reviewed by the physician and the patient. The patients were given cognitive oriented counseling, advice, additional information and reassuring. Appropriate coping strategies were proposed. Further intervention regarding symptomatic treatment, radiological examination or sick-leave was given if needed.

### Follow up

All randomized patients were examined 3 and 12 months after the head injury. The interviews comprised questions about the classical post concussion symptoms headache, dizziness, irritability and subjective personal changes. Thereafter patients underwent a comprehensive neurological examination. To detect depression, all patients were assessed with the Beck Depression Inventory, a self-report rating inventory measuring characteristic symptoms and severity of depression [20]. To examine changes in daytime vigilance and fatigue we used the Epworth Sleepiness Scale and the Fatigue Severity Scale respectively [21,22].

We assessed quality of life with the SF-36, a questionnaire aimed at capturing the relative impact of disease on physical and social functioning, role activities due to physical/emotional functioning, bodily pain, vitality (energy and fatigue), mental health and general health perception [23].

### Ethics

The study was approved by the Regional Ethics Committee. All participants gave written informed consent before study inclusion.

### Statistics

Continuous variables were normally distributed. Means were analyzed with student's t-test for independent or paired samples, respectively. Comparisons of proportions were performed using the chi-squared test for trends, or the Fishers exact test for small samples (expected count in one cell ≤ 5). Probability values are two-tailed, and p-values < 0.05 were considered significant. We used the Statistical Package for the Social Sciences (SPSS Inc., Chigaco, Illinois, release 14.0) for all analyses.

## Results

Table 1 shows baseline characteristics. There were no statistically significant differences between the intervention group and the control group. Table 2 shows outcomes at 3 months and change from three to twelve months. There were small improvements in the scores for depression, fatigue, quality of life and sleep disturbances between three and twelve months. The proportion reporting symptoms was also reduced from three to twelve months. The improvement was statistically significant only for the fatigue score, headache and irritability.

**Table 1:** Clinical characteristics of the study groups

Characteristic	Intervention group (n = 22)	Control group (n = 28)	p-value
Mean age (range)	40 (16-65)	43 (15-83)	n.s.
Males (percent)	13 (59%)	19 (67%)	n.s.
GCS score			
13	2	4	n.s.
14	4	9	
15	16	15	
CT examination (percent)	20 (91%)	25 (89%)	n.s.
CT verified traumatic intracranial injury (percent)	2 (9%)	4 (14%)	n.s.
Admitted to hospital (percent)	20 (91%)	22 (79%)	n.s.

GCS: Glasgow Coma Scale; CT: computerised tomography.

**Table 2:** Outcomes at 3 and 12 months after mild head injury in 50 patients

Outcome measure	3 months	12 months	p-value
BDI score (mean (95% C.I.))	7.5 (5.6 - 9.4)	6.8 (5.0-8.5)	0.33
FSS score (mean (95% C.I.))	36.0 (31.4 - 40.7)	32.5 (28.7-36.2)	0.04
SF-36 score (mean (95% C.I.))	104.4 (102.1 - 106.8)	106.3 (104.6-107.9)	0.09
ESS score (mean (95% C.I.))	7.7 (6.5 - 8.4)	7.2 (6.2-8.2)	0.28
Headache (percent)	15 (30%)	7 (14%)	0.04
Vertigo (percent)	12 (24%)	8 (16%)	0.39
Personality changes (percent)	4 (8%)	3 (6%)	1.00
Irritability (percent)	9 (18%)	2 (4%)	0.04
Symptomatic treatment (percent)	8 (16%)	6 (12%)	0.71

C.I.: confidence interval; BDI: Beck Depression Inventory; FSS: Fatigue Severity Scale; ESS: Epworth Sleepiness Scale.

Table 3 compares the intervention group and control group at 3 and 12 months follow up. There were no differences between the two groups neither at three nor at twelve months.

**Table 3:** Comparison of outcomes in the intervention group and the control group 3 and 12 months after mild head injury in 50 patients

	3 months			12 months		
	
Outcome measure	Intervention group (n = 22)	Control group (n = 28)	p-value	Intervention group (n = 22)	Control group (n = 28)	p-value
BDI score (mean (95% C.I.))	7.2 (4.7 - 9.7)	7.8 (4.9 - 10.7)	0.77	6.6 (4.1 - 9.1)	6.9 (4.3 - 9.5)	0.85
FSS score (mean (95% C.I.))	35.9 (28.4 - 43.5)	36.1 (30.0 - 42.3)	0.96	30.4 (24.8 - 36.0)	34.1 (28.8 - 39.3)	0.33
SF-36 score (mean (95% C.I.))	105.6 (101.5 - 109.6)	103.5 (100.6 - 106.4)	0.39	107.2 (104.8 - 109.5)	105.5 (103.1 - 107.9)	0.33
ESS score (mean (95% C.I.))	8.4 (6.6 - 10.1)	7.1 (5.5 - 8.8)	0.29	7.5 (5.9 - 9.0)	7.0 (5.6 - 8.4)	0.65
Headache (percent)	9 (41%)	5 (21%)	0.21	3 (14%)	4 (14%)	0.64
Vertigo (percent)	5 (23%)	7 (25%)	0.56	5 (23%)	3 (11%)	0.22
Personality changes (percent)	0 (0%)	4 (14%)	0.09	1 (5%)	2 (7%)	0.59
Irritability (percent)	4 (18%)	5 (18%)	0.63	1 (5%)	1 (4%)	0.69
Symptomatic treatment (percent)	2 (9%)	6 (21%)	0.22	4 (18%)	2 (7%)	0.23

BDI: Beck Depression Inventory; C.I.: confidence interval; FSS: Fatigue Severity Scale; ESS: Epworth Sleepiness Scale.

## Discussion

### Principal findings

This study shows that a significant proportion of the patients suffered from post concussion symptoms 3 months after the head injury, and that the symptoms improved from three to twelve months follow up. The main finding in the present study is that there was no effect on outcomes from an educational intervention two weeks after the injury.

### Strengths and weaknesses of the present study

We report results from a prospective randomized study but the high drop-out rate (85%) is a substantial limitation in our study. Drop out is, however, a common methodological problem in follow up studies after head injury. Previous randomized studies with designs comparable to ours report drop out rates between 10 and 59% [13-17]. Wade and co workers [13] used repeated telephone calls and other efforts to maximize follow up, but experienced a drop out rate of 59%. They considered this as a reflection of clinical realities, caused by low motivation among subjects with no or minor complaints, and speculated that patients completing the study had more complaints than the drop outs.

We relied on postal invitation only. A more aggressive strategy would have decreased the drop out rate, but probably not eliminated the problem. The proportion of patients with GCS scores 13 or 14 was higher in the control group compared to the intervention group, but there were no statistically significant differences between the groups, indicating that a comparison for evaluation of the intervention is relevant. Despite this, a real outcome difference between the groups may have been overseen as a result from the significant drop out (type II error).

It is another significant problem that there is no standard for outcome evaluation after head injury. The different studies referred to in this paper all employed different symptom scales and questionnaires for outcome assessment. Accordingly, direct comparison between the studies implies uncertainties.

Future studies should search to develop effective strategies for increasing follow up rates and standardization of outcome measures after head injury.

### Relation to other studies

The literature reports two other randomized studies comparing a single early intervention with a control group. Elgmark Andersson et al.[17] studied 395 patients with mild traumatic brain injury and allocated 264 to an early intervention and 131 to a control group. The intervention group was contacted by telephone after three weeks and patients with complaints were offered an outpatient consultation with information, counseling encouragement and assessment for the need for pharmaceutical therapy. At follow up after 12 months, there were no group differences in the rate of PCS or in life satisfaction. Ponsford and co-workers [15] included 202 patients with mild head injury. They assigned 79 to an early (five to seven days) intervention including education on common complaints and coping strategies, while 123 patients received no treatment. Patients in the intervention group had a moderate, but statistically significant reduction in symptom score at three months follow up. Neuropsychological tests showed no group differences. Taken together, the three studies by Elgmark Andersson and co workers, Ponsford and co-workers and our group indicate no or a very moderate effect from an early single educational intervention.

Wade and co-workers [13] studied 314 patients with head injuries of all severity grades. They randomized 184 to an intervention group and 130 to a control group. The intervention group received a comprehensive follow up consisting of information and advice on coping strategies, and repeated consultations including continuing advice, cognitive psychotherapy and referral to other specialists. At six months follow up, patients in the intervention group reported significantly less disruption of social activities and fewer symptoms. The effect of the intervention was most pronounced in the mild and moderately head injured groups. This study suggests that a more extensive intervention may be more effective than a single educational intervention. On the other hand, Paniak and co-workers [14] studied 105 adults with mild traumatic head injury. They randomly assigned the patients to a single session treatment similar to those in the previously mentioned studies, or to a treatment as needed group involving a comprehensive service from neuropsychologists and physiotherapists. In contrast to Wade and co workers study, they found no benefit from the comprehensive approach after 3 and 12 months. DeKruijk and co-workers [16] randomized 107 patients with mild traumatic brain injury to bed rest for six days (n = 53) or no bed rest (n = 54). There were no differences between the groups at three and six months after the injury.

## Conclusions

In the present study, there was no effect on outcomes from an early educational intervention two weeks after minimal, mild or moderate head injury. This is in accordance with one other study with a similar design, while a third study found a small, but statistically significant effect from such an intervention. It has been suggested that a more extensive intervention may be more effective, but the evidence on this is conflicting.

## Competing interests

The authors declare that they have no competing interests.

## Authors' contributions

BH and RB designed the study. BH and RB conducted data collection. All authors participated in data interpretation, literature research and preparation of the manuscript. All authors read and approved the final manuscript.
